# Supplementary material for: The causal relationship between the human gut microbiota and pyogenic arthritis: a Mendelian randomization study
Source: Front Cell Infect Microbiol. 2024 Nov 26;14:1452480. doi: 10.3389/fcimb.2024.1452480 (PMC11629706; doi:10.3389/fcimb.2024.1452480)
Supplement: Supplementary file 1 [file DataSheet1.zip › mendelian test/ebi-a-GCST90027698.csv_leave_one_out.pdf]

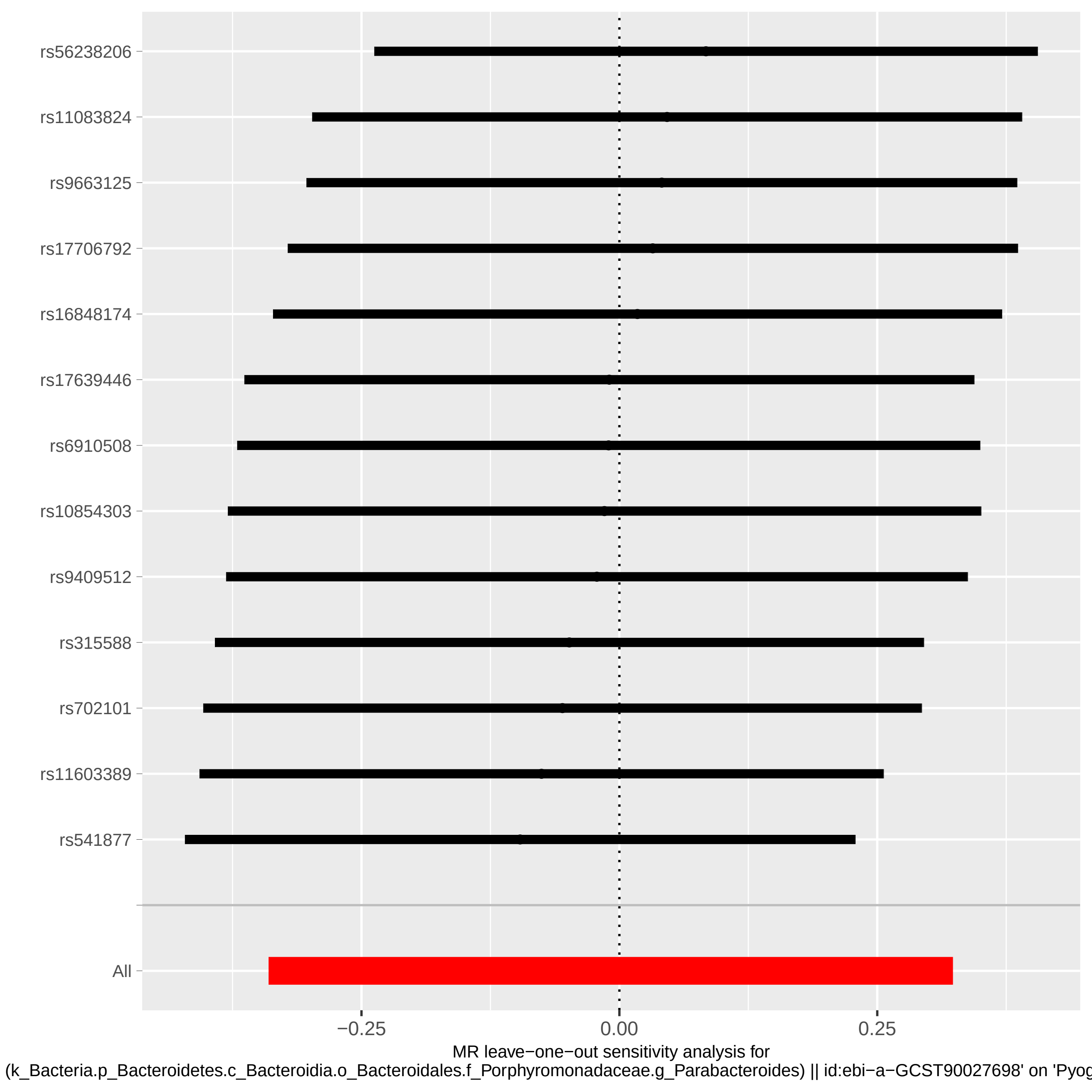

rs56238206
rs11083824
rs9663125
rs17706792
rs16848174
rs17639446
rs6910508
rs10854303
rs9409512
rs315588
rs702101
rs11603389
rs541877
All
−0.25
0.00
0.25
MR leave−one−out sensitivity analysis for
(k_Bacteria.p_Bacteroidetes.c_Bacteroidia.o_Bacteroidales.f_Porphyromonadaceae.g_Parabacteroides) || id:ebi−a−GCST90027698' on 'Pyog
